# Supplementary material for: Expression of Human Mutant Preproinsulins Induced Unfolded Protein Response, Gadd45 Expression, JAK-STAT Activation, and Growth Inhibition in Drosophila
Source: Int J Mol Sci. 2021 Nov 7;22(21):12038. doi: 10.3390/ijms222112038 (PMC8584581; doi:10.3390/ijms222112038)
Supplement: Supplementary file 1 [file ijms-22-12038-s001.zip › ijms-1416234-supplementary.pdf]

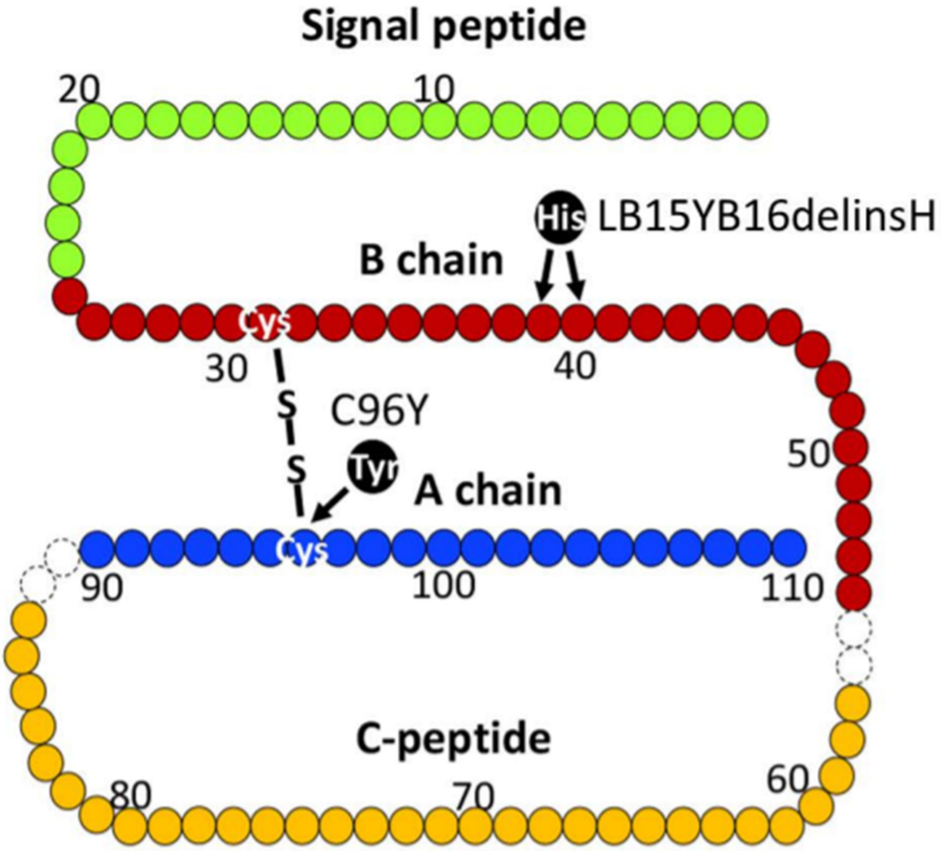

Figure S1. Expression of *Drosophila* insulin-like peptide 2 (Dilp2), human normal preproinsulin, or human mutant preproinsulin carrying a mutation derived from a PNDM model in *Drosophila* Insulin Producing Cells (IPCs), accumulation in starvation, and a release from the cells after re-feeding.

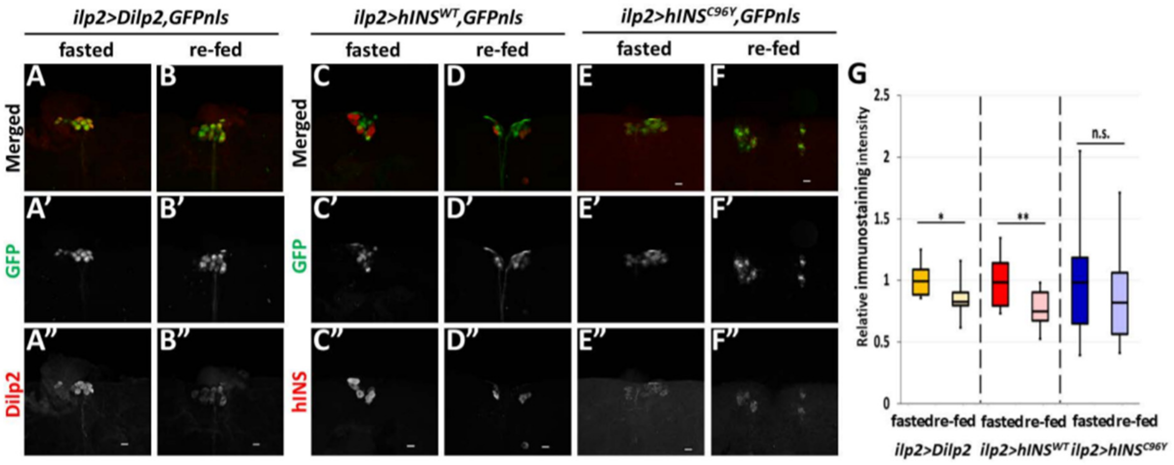

Figure S2. Expression of *Drosophila* insulin-like peptide 2 (Dilp2), human normal preproinsulin , or human mutant preproinsulin carrying a mutation derived from a PNDM model in *Drosophila* Insulin Producing Cells (IPCs), accumulation in starvation, and a release from the cells after re-feeding. (**A**–**F**) Immunostaining of larval brains harboring ectopic expression of GFPnls and Drosophila insulin-like peptide 2 (Dilp2) (*ilp2* > *ilp2, GFPnls*) (**A**,**B**), human normal preproinsulin (*ilp2* > $hINS^{WT}$, *GFPnls*) (**C**,**D**), or mutant preproinsulin, $hINS^{C96Y}$ (*ilp2* > $hINS^{C96Y}$, *GFPnls*) (**E**,**F**) with the relevant anti-Dlp2 or anti-human insulin antibody. Fluorescence microscopy images of IPCs in adults after 12 hrs starvation (**A**,**C**,**E**), and subsequently re-feeding the fly diet for 2 hrs (**B**,**D**,**F**). GFP fluorescence to label the IPCs (green in **A**–**D**, white in **A'**–**D'**), immunostaining signal (red in **A**–**D**, white in **A''**–**D''**). Bar: 10μm. (**G**) Quantification of GFP fluorescence intensity in the IPCs harboring ectopic expression of Dilp2, human normal preproinsulin, and the mutant preproinsulin, $hINS^{C96Y}$ in the adults after the starvation (fasted), and subsequent re-feeding (re-feeding). A relative intensity of immunofluorescence was calculated and normalized to the value of the IPCs in the fasted adults, which was set as 1.0. *: $p < 0.05$, **: $p < 0.01$, Student's *t*-test.
